# The approach of Iranian clinical pharmacists in the treatment of dyslipidemia based on international guidelines

**DOI:** 10.34172/jcvtr.2023.31585

**Published:** 2023-03-16

**Authors:** Mehrzad Mirshekari, Nooshin Shirzad, Mahboobeh Hemmatabadi, Soha Namazi

**Affiliations:** ^1^Clinical Pharmacy Department, School of Pharmacy, Tehran University of Medical Sciences, Tehran, Iran; ^2^Endocrinology and Metabolism Research Center, Endocrinology and Metabolism Clinical Sciences Institute, Tehran University of Medical Sciences, Tehran, Iran; ^3^Research Center for Rational Use of Drugs, Tehran University of Medical Science, Tehran, Iran

**Keywords:** Dyslipidemia, Clinical Pharmacist, Guideline

## Abstract

***Introduction:*** Optimal treatment of dyslipidemia is a top priority in the prevention of cardiovascular diseases. For this purpose, clinicians in Iran usually refer to four current international guidelines. The aim of this study was to assess the approach of Iranian clinical pharmacists in the treatment of dyslipidemia based on international guidelines.

***Methods:*** A structured questionnaire was prepared. Questions (n=24) included the demographics (n=7), dyslipidemia references (n=3), dyslipidemia general knowledge of respondents (n=10), and questions (n=4) designed based on the difference among the latest version of guidelines participants stated they use in their practice. After validity conformation, the questionnaire was distributed to 120 clinical pharmacists, electronically from May to August 2021.

***Results:*** Response rate was 77.5% (n=93). The majority of participants (80.6%, n=75) claimed to have used the 2018 ACC/AHA guideline. The Median (interquartile range [IQR]) score of the general knowledge questions was 5.0 (2.0) out of 10. The Median (IQR) score of questions designed based on the difference among guidelines was calculated 3(1) out of 4. There was no significant (*P*=0.25) difference in score among participants according to their guideline selection. Moreover, the gender and length of experience as a clinical pharmacist had no significant (*P*>0.05) effect on the score of participants.

***Conclusion:*** In this study, Iranian clinical pharmacists answered half of the dyslipidemia general knowledge questions correctly. Also, Participants were up-to-date on 75% of the questions designed based on the latest version of the guideline they had been using in their practice.

## Introduction

 According to the World Health Organization (WHO) report, cardiovascular diseases (CVDs) are the leading cause of death worldwide, with an estimated 17.9 million deaths annually. Dyslipidemia is one of the main risk factors for CVDs.^1^ According to the American Heart Association (AHA) report, 93 million American adults have total cholesterol serum levels of more than 200 mg/dl.^2^ A study evaluated dyslipidemia prevalence in 31050 Iranian adults in 2016. Its results showed that 80% of these individuals had at least one lipid abnormality. Although 74.2% of these population were aware of their hypercholesterolemia, only 44.2% were on statins.^3^ Furthermore, studies reported that the prevalence of those who achieved the desired lipid level was low among those who were on lipid-lowering drugs.^4-6^ As stated by a study in Greece, 66.6% of statin-treated patients are not reaching goal blood lipid levels.^4^

 Despite the plenty of evidence proving ways to reduce atherosclerosis induced by dyslipidemia, there are still gaps between the agreed standards of care and actual care.^7^ Evidence-based guidelines serve as a suitable decision-making tool to minimize variation in practice and provide a more rational basis for referral. While these guidelines do not replace clinical judgment, they help clinicians achieve better outcomes for patients.^8^ Several studies evaluated clinicians’ knowledge about guidelines. Zaitoun et al reported inadequate knowledge of physicians and clinical pharmacists about American College of Cardiology and American Heart Association (ACC/AHA) guidelines for dyslipidemia management.^9^ A study in Croatia showed only 50% of physicians used Joint European guidelines and on average, their knowledge of guidelines was insufficient.^10^ Another study evaluated the relationship between dyslipidemia knowledge of Chinese physicians and the low-density lipoprotein cholesterol (LDL-C) goal achievement in patient with dyslipidemia. Results revealed the LDL-C goal achievement rate was significantly (*P* < 0.0001) higher in patients whose physicians used the recommendations of the 2007 Chinese guidelines.^11^

 Therefore, due to the high prevalence of dyslipidemia in Iranian people, treatment of patients based on dyslipidemia guidelines can lead to a lower rate of CVDs, patient’s disabilities, and socioeconomic costs.^3^

 Owing to the lack of national lipid guidelines at the time of this publication, clinicians in Iran tended to established international guidelines to treat dyslipidemia. These guidelines include 2019 European Society of Cardiology and the European Atherosclerosis Society (ESC/EAS), 2018 ACC/AHA, 2003 National Cholesterol Education Program-Adult Treatment Panel III (NCEP-ATP III), and 2020 American Association of Clinical Endocrinologists and American College of Endocrinology (AACE/ACE). These guidelines have major differences, such as statin-benefited population and LDL-C serum goal in diverse patients.^12^ Clinical pharmacists in Iran have an important role in the treatment of dyslipidemic patients. Based on educational program, they update their knowledge by studying the latest guidelines recommendations and help physicians to improve the pharmacotherapy of disease. According to our search in databases (e.g., Google scholar, PubMed, Scopus) no published studies have been found that assessed the approach of Iranian clinical pharmacists in the treatment of dyslipidemia based on multiple international guidelines (2019 ESC/EAS, 2018 ACC/AHA, 2003 NCEP-ATP III and 2020 AACE/ACE). This study aimed to evaluate the level of knowledge of clinical pharmacists to improve the evidence-based management of dyslipidemia.

## Materials and Methods

 This cross-sectional study was conducted from May to August 2021 among Iranian clinical pharmacists. To collect the data, a structured questionnaire consisting of 24 questions was prepared in EPOLL software and sent to the participants via social media (Appendix 1). All participants were assured that all data would be kept confidential. The questions were designed by four academic members of Tehran University of Medical Sciences (TUMS), including two clinical pharmacists and two endocrinologists. Inclusion criteria were Iranian board-certified pharmacotherapy specialists who were satisfied to participate in the study. The exclusion criteria were participants who did not complete the questionnaire. The questionnaire included 7 questions about the demographics and professional characteristics of the participant, 3 questions regarding references used in the treatment of dyslipidemia, 10 questions about general dyslipidemias knowledge of respondents, and 4 questions designed based on differences among the latest version of guidelines that participants use in their practice. Generally, questions were designed based on participant’s knowledge about LDL-C and triglyceride (TG) treatment goals, statins benefited population, statins optimal dose in different medical conditions like stroke, diabetes, chronic kidney disease, and myocardial infarction, statins use in pregnancy, hypertriglyceridemia treatment and statin cost-effective.

 To determine the validity of the study instrument, eight clinical pharmacists and endocrinologist selected one of the three-degree range of “not necessary, useful but not essential, essential”for each question. Then the content validity ratio (CVR) was calculated. The formula of CVR is (Ne - N/2)/ (N/2), in which the Ne represents the essential number of clinical pharmacists and endocrinologists and N is the total number of clinical pharmacists and endocrinologists.^13^ CVR was obtained more than 0.75 for all questions. Also, Cronbach’s alpha was used to measure internal consistency which was computed by 0.9. In this study, one point is considered for each correct answer and zero for each incorrect answer. A score out of 4 was calculated to evaluate how closely their responses matched the lipid guidelines they stated. Also, a score out of 10 was calculated to assess the general knowledge of participants about dyslipidemia treatment. The participants’ knowledge was estimated by calculating the Median (interquartile range (IQR)). The sample size was estimated at 90 by the n = Z^2^1- α/2 p (1-p)/d^2^ formula. In this formula, n = sample size, z = level of confidence according to the standard normal distribution (for a level of confidence of 95%, z = 1.96, α (error level) = 5%, p (estimated population proportion presents the characteristic) = 0.5 and d (tolerated margin of error) = 0.05. Statistical analysis was performed using SPSS software version 26.Statistical tests were used to compare the knowledge of clinical pharmacists and the type of guidelines. The Kolmogorov-Smirnov test was utilized to determine whether sample data is normally distributed and the Kruskal–Wallis test was to distinguish whether there are statistically significant differences among groups. Also, the Mann-Whitney U test was used to compare the knowledge of males and females. The Kruskal–Wallis test was used to compare participants’ knowledge and length of experience as a clinical pharmacist (respondents were categorized in to 3 groups including: 0-5, 6-10, and 11-20 years of experience). A level of P < 0.05 was considered statistically significant.

## Results

 The questionnaire was distributed among 120 clinical pharmacists, and the response rate was 77.5% (n = 93). Table 1 shows the demographic data. It shows that most of the participants (61.3%, n = 57) were female, and 73.1% (n = 68) of respondents were between 25 and 35 years old. The mean age of participants was 33.5 ± 1.2 years. Although participants worked in different cities, 74.2% (n = 69) of them worked in Tehran (the capital of Iran) in the 5 last years. Data analysis exhibited that 66.7% (n = 62) and 78.5% (n = 73) of respondents had the length of work experience between 0-5 years as a general pharmacist and clinical pharmacist, respectively. Also, more than a third of the participants (39.8%, n = 37) worked in several centers. Moreover, 33.3% (n = 31) of respondents were academic members, and 6.4% (n = 2) only worked at the university, not in clinical centers.

**Table 1:** Demographics and individual characteristics of participants (n = 93).

**Age (year)**	**N (%)**
25-30	35(37.6%)
31-35	33(35.5%)
36-40	15(16.1%)
41-50	8(8.6%)
> 50	2(2.2%)
**Gender**	**N (%)**
Male	36(38.7%)
Female	57(61.3%)
**City of work**	**N (%)**
Tehran	69(74.2%)
Sari	10(10.8%)
Shiraz	4(4.3%)
Esfahan	4(4.3%)
Others	6(6.6%)
**Academic member**	**N (%)**
Yes	31(33.3%)
No	62(66.7%)
**Workplace**	**N (%)**
Multicenter^*^	37(39.8%)
Faculty	15(16.1%)
Public hospital	32(34.4%)
Private hospital	1(1.1%)
Other centers	8(8.6%)
**Length of experience (year)** **as general/clinical pharmacist**	**N (%)**
0-5	62 (66.7%)**/**73(78.5%)
6-10	22 (23.7%)**/**15(16.1%)
11-20	9 (9.7%)**/**5(5.4%)

*Participants who work simultaneously in several centers such as faculty and hospital.

**Figure 1:**
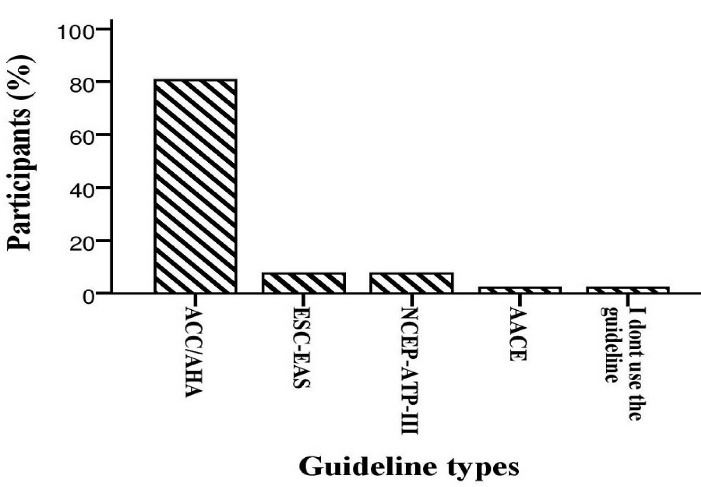
Frequency of participants’ guideline selection in the treatment of dyslipidemia. Most participants (80.6%, n = 75) selected the 2018 ACC/AHA guideline. ACC/AHA: American College of Cardiology and American Heart Association, ESC/EAS: European Society of Cardiology and the European Atherosclerosis Society, NCEP-ATP III: National Cholesterol Education Program-Adult Treatment Panel III, AACE: American Association of Clinical Endocrinologists.

**Figure 2:**
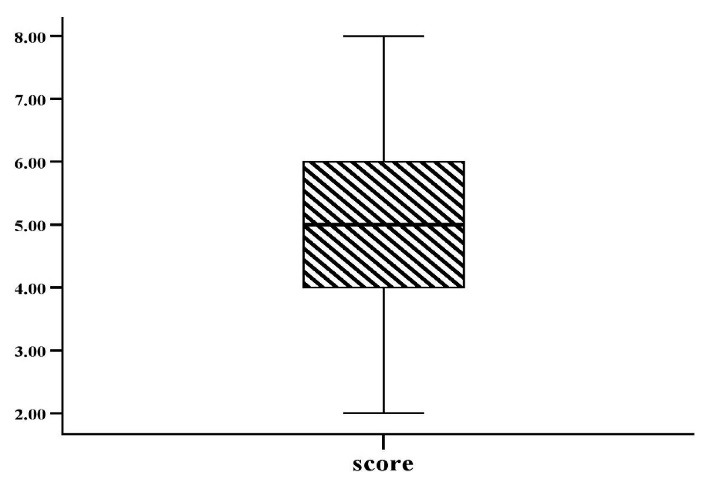
Participant’s score of general knowledge questions in dyslipidemia treatment. A score out of 10 was calculated for each participant. Participants scored between 2 and 8. The Median (IQR) score was calculated as5.0 (2.0). IQR: interquartile range.

Figure 1 shows the selection of different guidelines in the treatment of dyslipidemia by participants. Most participants (80.6%, n = 75) selected the 2018 ACC/AHA guideline. Figure 2 shows participants’ scores of general knowledge questions in the treatment of dyslipidemia. The Median (IQR) of the general knowledge question’s score was calculated to be 5.0 (2.0) out of 10.

**Figure 3:**
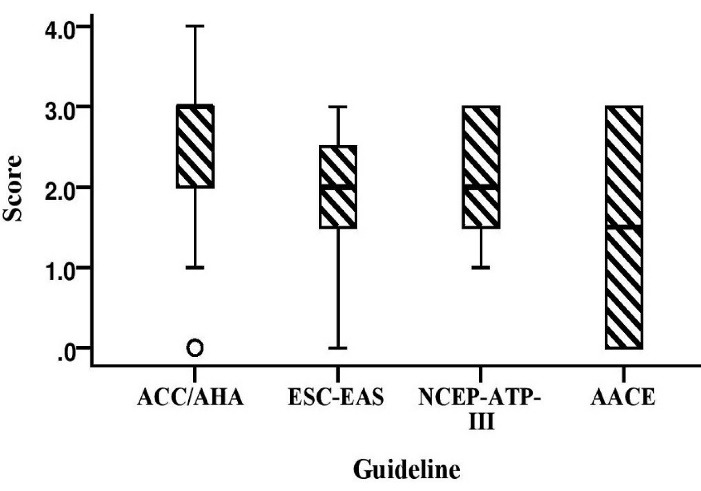
The relationship between participant’s guideline selection and the score they achieved after responding to four questions designed based on the differences among guidelines. A score out of 4 was calculated for each participant. There was no significant difference (P = 0.25) between groups. American College of Cardiology and American Heart Association, ESC/EAS: European Society of Cardiology and the European Atherosclerosis Society, NCEP-ATP III: National Cholesterol Education Program-Adult Treatment Panel III, AACE: American Association of Clinical Endocrinologists.

Figure 3 shows the relationship between participants› guideline selection and the score they achieved after responding to four questions designed based on the differences among guidelines. The results showed that 11.0% (n = 10), 41.8% (n = 38), 30.8% (n = 28), 12.1% (n = 11), and 4.3% (n = 4) of the participants received scores of 4, 3, 2, 1 and zero, respectively. The Median (IQR) scores of all participants were 3 (1) out of 4 and 75.0% of total score was achieved. The Median (IQR) scores were calculated 3.0 (1.0), 2.0 (2.0), 2.0 (2.0), and 1.5 for 2018 ACC/AHA, 2019 ESC/EAS, 2003 NCEP-ATP III, and 2020 AACE/ACE, respectively There was no significant difference between the groups (*P* = 0.25).

 Analysis exhibited there were no significant differences (for general knowledge (*P* = 0.74) and guideline-based questions (*P* = 0.75)) between the knowledge of males and females. Also, there was no significant differences in general knowledge (*P* = 0.14) and guideline-based questions (*P* = 0.25) between knowledge and the length of experience of participants as clinical pharmacists.

## Discussion

 This study assessed the approach of Iranian clinical pharmacists in the management of dyslipidemia based on international guidelines. According to the results, the Median score of dyslipidemia general knowledge questions was 5 out of 10.Indeed, these findings indicate the high importance of education in pharmacy. Similarly, a cross-sectional study evaluated the awareness of 77 physicians and clinical pharmacists about the 2013 ACC/AHA guideline for dyslipidemia management in Saudi Arabia. The Median score of participants was calculated 4 out of 10, and inadequate knowledge of the major changes in ACC/AHA guideline for the management of dyslipidemia has been reported. Likewise, there was no significant difference (*P* = 0.88) in knowledge between clinical pharmacists and physicians. Furthermore, this study showed that female practitioners’ level of knowledge was superior to their male peers (*P* < 0.03).^9^ whereas our study results exhibited no significant (*P* = 0.80) difference in knowledge of males and females.

 In a study by John d et al the knowledge, awareness, and level of agreement of clinical pharmacists with the 2013 ACC/AHA cholesterol guideline were evaluated through a valid questionnaire. They reported that although most respondents had studied this guideline, some knowledge gaps were identified, including understanding the four outcomes of the atherosclerotic cardiovascular disease (ASCVD) risk estimator and knowing the difference between the Framingham Risk Score and ASCVD risk estimator.^14^ Another study in Malaysia found that 70.2% of 466 postgraduate primary care trainees received at least a 70% knowledge about national lipid guidelines. Also, there was a positive notable (*P* < 0.001) relation between awareness and the use of the guideline with knowledge score.^15^ In the present study, participants correctly answered 75% of the questions designed based on the latest version of the guideline they stated they use in their practice. Also, there was no significant (*P* = 0.25) difference among respondents’ knowledge in the groups (2018 ACC/AHA, 2019 ESC/EAS, 2003 NCEP-ATP III, and 2020 AACE/ACE). In addition, %89.2 of participants (n = 83) used cardiovascular risk estimator in their practice. Because clinical pharmacists advise physicians, especially for complicated disease, they should always be up to date on the use of new guidelines, as confirmed by the results of our study.

 Alhajji et al evaluated the Kuwaiti physicians’ adherence to lipid guidelines via a valid questionnaire. The findings showed that more than 90% (n = 251) of them stated to use guideline. Additionally, the most common lipid guideline used by these physicians (85%, n = 237) was a guideline on the treatment of blood cholesterol published by the ACC/AHA. Also, lack of access and time limitations were the most barrier to using guidelines.^16^ Similarly, in our study, most participants (61.3%, n = 57) selected ACC/AHA in their practice for dyslipidemia management. Moreover, only 2.1% (n = 2) of participants did not use the guidelines due to a lack of access and trust in their clinical experience. Several studies reported the gap between evidence-based guidelines recommendations and their clinical experience that included: lack of access to the guidelines, lack of agreement on guidelines recommendations, lack of expected benefits and limited patients’ involvement and time.^17-19^

 Analysis of results demonstrated respondents manage dyslipidemia based on different guidelines or clinical experience, while the evidence behind these guidelines is generally based on data from Western populations, and their application to the Iranian people has not been largely tested and compared. This finding was consistent with the results of Alshamiri et al that practitioners within Asia and the Middle East continue to rely on international evidence despite population differences in lipid phenotypes and CVD risk factors.^20^ Therefore, efforts should be made to improve education and preparation of local guidelines to reduce the gap between the approaches of different clinicians in the treatment of dyslipidemia. Also, the outcome of the national guidelines based on practice should be evaluated and compared with the international guidelines.

 This study had some limitations. First, due to the limited number of clinical pharmacists in Iran, the sample size was small. Additionally, participants worked in different wards of the hospital and many of them did not manage dyslipidemic patients. Therefore, the participants may not have enough knowledge to fill out the questionnaire, correctly. In the end, most of the participants (74.2%, n = 69) were working in Tehran, and it was not possible to compare the knowledge of participants based on their site of practice.

## Conclusion

 In conclusion, Iranian clinical pharmacists answered half of the dyslipidemia general knowledge questions correctly. The 2018 AHA/ACC was the most widely used of the guidelines. Moreover, participants were up-to-date on 75% of the questions based on the latest version of the guideline used in their practice.

## Acknowledgments

 We would like to express our very great appreciation to all the study participants for the substantial time and effort given to the present investigation. Also, we acknowledge the generous financial support of the Research Center for Rational Use of Drugs, TUMS, Tehran, Iran by grant No. 1400-1-207-52848.

## Competing Interest

 The authors have no conflict of interest to declare.

## Ethical Approval

 This study was approved by the ethics committee of TUMS and received ethical Code number IR.TUMS.TIPS.REC.1400.088.

## Funding

 This study was financially supported by grant No. 1400-1-207-52848 from the Research Center for Rational Use of Drugs, Tehran University of Medical Sciences, Tehran, Iran.

## Appendix

**Appendix 1:** Evaluation of the Approach of Iranian Clinical Pharmacists in the Treatment of Dyslipidemia based on International Guidelines. The frequency and percentage of participants who answered each question correctly are stated in the dyslipidemia knowledge section.

** Demographics and professional characteristics questions:**

- Gender:

o Male
o Female

- Age: …
- Years of experience as a general pharmacist: …
- Years of experience as a clinical pharmacist: …
- The name of city that you worked in the last 5 years: …
- Your workplace (you can select more than one option):

o Public hospital
o Private hospital
o Faculty
o Multicenter
o Other centers

- Academic member:

o Yes
o No

** Dyslipidemia references questions:**

- Which one is the main basis of your Decision-making in the treatment of dyslipidemia:

o International guidelines
o Scientific seminars and retraining courses
o Personal experience

- Which guideline do you use in your practice? (If you use a guideline):

o American College of Cardiology and the American Heart Association (ACC/AHA)
o American Association of Clinical Endocrinologists (AACE)
o European Society of Cardiology and the European Atherosclerosis Society (ESC/EAS)
o National Cholesterol Education Program Adult Treatment Panel III (NCEPT ATP III)
o I don’t use the guideline

- What’s your reason in the previous question if you chose “I don’t use the guideline”:

o Lack of access to guidelines
o Extensive guidelines and lack of time
o Trust in clinical experience
o Quick changes to guidelines

** Dyslipidemia knowledge questions:**

- Which one is correct, according to the definition of hypertriglyceridemia in adults (mg/dl): ***(%39.8, n*** ***=*** ***37)***

o TG > 149
o TG > 199
o TG > 499

- Which one is the high LDL-C level in adults, according to international guidelines (mg/dl): ***(76.3%, n*** ***=*** ***71)***

o LDL-C > 100
o LDL-C > 160
o It varies from person to person depending on the risk factors

- Which option is acceptable based on dyslipidemia screening: ***(%18.3, n*** ***=*** ***17)***

o Fasting status is required
o Non-fasting status is acceptable

- Do you calculate the score of cardiovascular risk factors to decide on treatment: ***(%89.2, n*** ***=*** ***83)***

o Yes
o No

- In which of the following options is it necessary to rule out secondary causes of hypertriglyceridemia (mg/dl): ***(%14.0, n*** ***=*** ***13)***

o TG > 150
o TG > 500
o TG > 1000

- A 48-year-old man with no history of cardiovascular disease or diabetes has a TG = 300 mg/dl. Which of the following do you choose for treatment: ***(%63.4, n*** ***=*** ***59)***

o Diet and exercise
o Diet, exercise, and fibrates
o Diet, exercise, and omega-3

- Which type of statins doesn’t require dose adjustment in patients with stage 4 and 5 chronic renal failure: *** (%61.3, n*** ***=*** ***57)***

o Atorvastatin
o Rosuvastatin
o Simvastatin

- A 30-year-old woman is taking atorvastatin and is planning to become pregnant. Which of the following do you recommend to the patient? ***(%44.1, n*** ***=*** ***41)***

o Discontinue atorvastatin at the onset of pregnancy
o Continue atorvastatin during pregnancy
o Discontinue atorvastatin 2 months before pregnancy

- A 40-year-old female head of household with no history of cardiovascular disease and diabetes presented with LDL-C = 200 mg/dl. Which treatment do you recommend? *** (%24.7, n*** ***=*** ***23)***

o Atorvastatin 20 mg/day
o Rosuvastatin 10 mg/day
o Atorvastatin 20 mg/day or rosuvastatin 10 mg/day

- In case of diabetes after starting treatment with statins, which of the following is appropriate? *** (%81.7, n*** ***=*** ***76)***

o Reduce the dose of the statin
o Discontinue treatment with statin
o Continue treatment with statin

** Dyslipidemia knowledge questions based on guideline selection of participants:**

- A 52-year-old woman with type 2 diabetes who has microalbuminuria in urinalysis. Which option is the LDL-C target (mg/dl): *** (%71.4, n*** ***=*** ***65)***

o LDL-C<55
o LDL-C<70
o LDL-C<100

- A 46-year-old man with a 10-year history of type 2 diabetes who has not yet received lipid lowering medication. The patient has a good lifestyle and has no other risk factors. In laboratory data, LDL-C = 70 mg/dl. Which treatment option do you choose: *** (%79.1, n*** ***=*** ***72)***

o Atorvastatin 20 mg/day
o Atorvastatin 40 mg/day
o No treatment is required

- A 59-year-old woman with a history of ischemic stroke and MI treated with rosuvastatin 40 mg/day. It currently has LDL-C = 65 mg/dl. Which option do you choose: *** (%82.5, n*** ***=*** ***75)***

o Replacement of rosuvastatin with atorvastatin 80 mg daily
o Add ezetimibe
o No other treatment is required

- Which of the following is a very high risk in the evaluation of lipid disorders? *** (%67.1, n*** ***=*** ***61)***

o A diabetic patient with a history of ischemic stroke
o A patient with chronic renal failure (GFR = 25 ml/min)
o Both options above

## References

[1] Du X, Su X, Zhang W, Yi S, Zhang G, Jiang S (2022). Progress, opportunities, and challenges of troponin analysis in the early diagnosis of cardiovascular diseases. Anal Chem.

[2] Virani SS, Alonso A, Benjamin EJ, Bittencourt MS, Callaway CW, Carson AP (2020). Heart disease and stroke statistics-2020 update: a report from the American Heart Association. Circulation.

[3] Aryan Z, Mahmoudi N, Sheidaei A, Rezaei S, Mahmoudi Z, Gohari K, et al. The prevalence, awareness, and treatment of lipid abnormalities in Iranian adults: surveillance of risk factors of noncommunicable diseases in Iran 2016. J Clin Lipidol 2018;12(6):1471-81.e4. 10.1016/j.jacl.2018.08.001. 30195823

[4] Liberopoulos E, Vlasserou F, Mitrogianni Z, Papageorgantas I, Elisaf M (2012). Prevalence and risk distribution of residual dyslipidemia in statin-treated patients in Greece. Angiology.

[5] Khovidhunkit W, Silaruks S, Chaithiraphan V, Ongphiphadhanakul B, Sritara P, Nimitphong H (2012). Prevalence of dyslipidemia and goal attainment after initiating lipid-modifying therapy: a Thai multicenter study. Angiology.

[6] Gitt AK, Drexel H, Feely J, Ferrières J, Gonzalez-Juanatey JR, Thomsen KK (2012). Persistent lipid abnormalities in statin-treated patients and predictors of LDL-cholesterol goal achievement in clinical practice in Europe and Canada. Eur J Prev Cardiol.

[7] Mantel-Teeuwisse AK, Verschuren WM, Klungel OH, Kromhout D, Lindemans AD, Avorn J (2003). Undertreatment of hypercholesterolaemia: a population-based study. Br J Clin Pharmacol.

[8] Bodenheimer T, Wagner EH, Grumbach K (2002). Improving primary care for patients with chronic illness. JAMA.

[9] Zaitoun MF, Iflaifel MH, Almulhim LA, Al-Ghamdi MA, Ibrahim YA (2019). Awareness of physicians and clinical pharmacists about ACC/AHA guidelines for dyslipidemia management: a cross-sectional study. J Pharm Bioallied Sci.

[10] Reiner Z, Sonicki Z, Tedeschi-Reiner E (2010). Physicians’ perception, knowledge and awareness of cardiovascular risk factors and adherence to prevention guidelines: the PERCRO-DOC survey. Atherosclerosis.

[11] Ding R, Ye P, Zhao S, Zhao D, Yan X, Dong Y (2017). Effect of physician characteristics and knowledge on the quality of dyslipidemia management and LDL-C target goal achievement in China: subgroup analysis of the Dyslipidemia International Study. J Glob Health.

[12] Bartlomiejczyk MA, Penson P, Banach M (2019). Worldwide dyslipidemia guidelines. Curr Cardiovasc Risk Rep.

[13] Zamanzadeh V, Ghahramanian A, Rassouli M, Abbaszadeh A, Alavi-Majd H, Nikanfar AR (2015). Design and implementation content validity study: development of an instrument for measuring patient-centered communication. J Caring Sci.

[14] Bucheit JD, Helsing H, Nadpara P, Virani SS, Dixon DL. Clinical pharmacist understanding of the 2013 American College of Cardiology/American Heart Association cholesterol guideline. J Clin Lipidol 2018;12(2):367-74.e3. 10.1016/j.jacl.2017.11.010. 29277495

[15] Said AH, Chia YC (2017). Awareness, knowledge and practice of dyslipidaemia management among postgraduate primary care trainees in Malaysia: a cross-sectional study. BMJ Open.

[16] Alhajji S, Mojiminiyi S (2020). Adherence to current lipid guidelines by physicians in Kuwait. Med Princ Pract.

[17] Vashitz G, Meyer J, Parmet Y, Henkin Y, Peleg R, Liebermann N (2011). Adherence by primary care physicians to guidelines for the clinical management of dyslipidemia. Isr Med Assoc J.

[18] Fürthauer J, Flamm M, Sönnichsen A (2013). Patient and physician related factors of adherence to evidence based guidelines in diabetes mellitus type 2, cardiovascular disease and prevention: a cross sectional study. BMC Fam Pract.

[19] Dallongeville J, Banegas JR, Tubach F, Guallar E, Borghi C, De Backer G (2012). Survey of physicians’ practices in the control of cardiovascular risk factors: the EURIKA study. Eur J Prev Cardiol.

[20] Alshamiri M, Ghanaim MMA, Barter P, Chang KC, Li JJ, Matawaran BJ (2018). Expert opinion on the applicability of dyslipidemia guidelines in Asia and the Middle East. Int J Gen Med.

